# The prevalence of edentulism and their related factors in Indonesia, 2014/15

**DOI:** 10.1186/s12903-018-0582-7

**Published:** 2018-07-03

**Authors:** Supa Pengpid, Karl Peltzer

**Affiliations:** 1 ASEAN Institute for Health Development, Mahidol University, Salaya, Thailand; 2 Department of Research & Innovation, University of Limpopo, Turfloop, South Africa; 3 HIV/AIDS/STIs and TB (HAST) Research Programme, Human Sciences Research Council, Private Bag X41, Pretoria, 0001 South Africa

**Keywords:** Edentulism, Nutrition, Health status, Tobacco use, Older adults, Indonesia

## Abstract

**Background:**

Little information exists about the loss of all one’s teeth (edentulism) among older adults in Indonesia. The aim of this study was to investigate the prevalence of edentulism and associated factors among older adults in Indonesia.

**Method:**

This study examines the self-reported prevalence of edentulism and associated factors among older adults (50 years and older) in a cross-sectional national study using the Indonesia Family Life Survey IFLS-5, 2014/15. The community-based study uses a multi-stage stratified sampling design to interview and assess household members, with a household response rate of over 90%.

**Results:**

The overall prevalence of edentulism was 7.2, 29.8% in 80 years and older and 11.8% in those with no formal education. In adjusted Poisson regression analysis, older age, living in five major island groups and having functional disability were associated with edentulism. In addition, among men, having quit and current tobacco use and among women, having low social capital were associated with edentulism. Further, in adjusted analysis, among men, edentulism was positively associated with hypertension and negatively associated with diabetes, and among women, edentulism was associated with functional disability.

**Conclusions:**

Results suggest that overall and/or among men or women that older age, living in five major island groups, having functional disability, tobacco quitters and users and those with low social capital was associated with edentulism. The identified associated factors of edentulism may be utilized in oral health programmes targeting older adults in Indonesia.

## Background

Edentulism (=having lost all of one’s natural teeth) is a significant public health problem globally because of its high prevalence (> 10% in individuals 50 years and older) and related disability [1, 2]. The disability-adjusted life-years (DALYs) of oral conditions dramatically increased between 1990 and 2015 (16.9 million years live with disability) [3]. Among oral disorders, edentulism accounted for more than one third (7.6 million DALYs) of the oral disorder disability burden, globally [4]. Monitoring the occurrence of edentulism [5] provides an indication of both population oral health and oral health care system response [6]. There is a lack of community data on oral health among older adults in low- and middle-income countries [7]. Based on data from the World Health Survey, conducted from 2002 to 2004, the prevalence of self-reported edentulism among persons 50 years and older in countries of the Southeast Asian region were 4.2% in Laos, 22.2% in Malaysia, 2.4% in Myanmar, 14.6% in the Philippines and 5.6% in Vietnam [1]. In a more recent study (2007–2008) among adults 50 years and older, the overall prevalence of edentulism was 11.7% in the six middle-income countries, with India, Mexico, and Russia having higher prevalence rates (16.3–21.7%) than China, Ghana, and South Africa (3.0–9.0%) [2, 8]. In Indonesians aged 65 years or over the prevalence of measured edentulism was 17.6% in 2007 [9].

Using a social determinants of health approach, factors associated with edentulism may include, in addition to caries and periodontal disease [10], sociodemographic factors such as increasing age, female gender, no or lower education, lower economic status and rural residence [2, 8, 11, 12]. Along with socioeconomic factors, health risk behaviours, such as smoking and former smoking remain strong predictors of edentulism [2, 11, 13–16]. Having chronic diseases [17], such as diabetes [18], having underweight [11], arthritis [2, 19], asthma [2, 11], depression [11, 17], reduced physical function [11], functional disability [2, 12], poor self-rated health [12, 17], lack of social support [17] and lack of social capital [20, 21] have also been found to be associated with edentulism. On the other hand, edentulism has been associated with poor health status [12, 22], insufficient fruit and vegetable consumption [22, 23], smoking [24], underweight, poor nutrition [25–27], overweight/obesity [27–29], hypertension [30, 31], angina [32], strokes [33], diabetes [25, 34], rheumatoid arthritis [25], asthma [25], and functional disability [35, 36].

There is a lack of studies on edentulism and associated factors in Indonesia [37, 38]. The aim of this study was to investigate the prevalence of edentulism and associated factors among older adults in Indonesia. The objectives include: (1) to estimate the prevalence of self-reported edentulism in older adults, and (2) to identify possible factors such as sociodemographics and health variables associated with edentulism in Indonesia.

## Method

### Study design and participants

Secondary data from the fifth wave of the “Indonesia Family Life Survey (IFLS-5) 2014-15” were analysed, being the only of the five surveys that assessed edentulism [39]. The IFLS-5 is a community survey interviewing household members (7994 50 years and older with complete endentulism measurements) selected by multistage stratified sampling representing 83% of the Indonesian population, with a household response rate of over 90%, more details [39, 40].

### Measures

*Edentulism* was assessed with the question, “Have you lost all your teeth?” (Yes, No) [39].

*Socio-demographic variables* included age, sex, education, area of residence, province, region and subjective socioeconomic background [39, 40].

*Social capital* was assessed with 4 items related to the past 12 month participation in “1) Community meeting, 2) Voluntary labour, 3), Programme to improve the village/neighbourhood, and 4) Religious activities.” Response options, were “yes” or “no” [39]. (Cronbach’s alpha 0.59). Those who scored 0 times with “yes” were considered as having low social capital.

*Self-reported health status* was measured with the item, “In general, how is our health?” (Responses ranged from 1 = Very healthy to 4 = Unhealthy) [39].

*Nutrition status.* Heights and weights were taken using standard procedures [39], and body mass index (BMI) calculated according to Asian criteria: “underweight (<18.5 kg/m^2^), normal weight (18.5 to <23.0 kg/m^2^), overweight (23.0 to <25.0 kg/m^2^) and 25+ kg/m^2^ as obesity” [41].

*Functional disability* was assessed by 5 items of Activity of Daily Living (ADL) and 6 items of Instrumental Activity of Daily Living (IADL) [42, 43]. A total functional disability score was calculated, with having no difficulty = 0 and one or more ADL/IADL items = 1.

*Tobacco use* was grouped into never, quitters and current tobacco users, following the questions of ever and current use of various tobacco products [39, 40].

*Inadequate fruit and vegetable consumption* was defined as eating less than 3 days a week fruits and less than daily vegetables, following questions on the number of days in the past week vegetables (green leafy vegetables and carrots) and fruits (banana, papaya and mango) had been consumed [39, 40].

*Chronic condition* was measured based on health care provider diagnosed “Diabetes or high blood sugar, High blood pressure, Heart attack, coronary heart disease, angina or other heart problems, Stroke, Arthritis and Asthma” (Yes, No) [39, 40].

### Hypertension

Average blood pressure was calculated arithmetically for three averaged measurements of systolic and diastolic blood pressure, assessed by using standard procedures [39]. Hypertension was defined as “SBP ≥140 mm Hg and/or DBP ≥90 mm Hg and/or current use of antihypertensive medication”. [44].

The *Centres for Epidemiologic Studies Depression Scale (CES-D:* 10 items) was used to assess depressive symptoms, and scores of 10 or more were classified as having depressive symptoms [45] (Cronbach’s alpha = 0.67).

### Data analysis

Descriptive statistics were used to describe the variables. Nonparametric tests were used for trend analyses across ordered groups. Associations between key outcomes of edentulism and sociodemographic and health variables were evaluated by calculating prevalence ratios (PR). Poisson regression was used for evaluation of the association of explanatory variables for the outcome of edentulism (binary dependent variable). The Pearson goodness-of-fit test was used. Logistic regression was used to determine the association of edentulism on various health outcome variables. The Hosmer-Lemeshow goodness-of-fit test was used to evaluate multivariable model fit. Potential multi-collinearity between variables was assessed with variance inflation factors, none of which exceeded critical value. *P* < 0.05 was considered significant. “Cross-section analysis weights were applied to correct both for sample attrition from 1993 to 2014, and then to correct for the fact that the IFLS-1 sample design included over-sampling in urban areas and off Java. The cross-section weights are matched to the 2014 Indonesian population, again in the 13 IFLS provinces, in order to make the attrition-adjusted IFLS sample representative of the 2014 Indonesian population in those provinces.” [39] Both the 95% confidence intervals and *P*-values were adjusted considering the survey design of the study. All analyses were done with STATA software version 13.0 (Stata Corporation, College Station, TX, USA).

## Results

### Sample characteristics

The total sample included 7994 older adults, 50 years and older (mean age 62.8 years, SD = 9.8, age range of 50–110 years) in Indonesia. The proportion of women was 51.9, 72.2% had no or elementary education, 44.2% described themselves as having medium economic status, 52.0% resided in urban areas, 58.1% were living in Java and 20.8% had low social capital. Regarding health status, 32.9% rated their health as unhealthy, and 31.3% had at least one functional disability. In all, 14.1% of participants were underweight and 21.3% overweight or obese, 33.5% infrequently consumed fruit and vegetables, 33.3% were current tobacco users and 9.8% quit tobacco use. In terms of chronic conditions, 6.3% had diabetes, 58.5% hypertension, 3.6% heart attack, angina or other heart problems, 2.9% had a stroke, 11.8% had arthritis, 11.8% asthma and 17.0% depressive symptoms. The overall prevalence of edentulism was 7.2, 7.6% among women and 6.8% among men, and among those 50 to 59 years 3.1% and those aged 80 years and older 29.8%, and with no education the prevalence of edentulism was 11.8% and those with higher education 2.1% (see Table 1).

**Table 1:** Sample characteristics and prevalence rate of edentulism among older adults in Indonesia

	Total sample	Prevalence rate of edentulism	
	*N* (%)	*n*	% (95% CI)^a^
Sociodemographics			
All	7994	685	7.2 (6.7, 7.8)
Age in years			
50–59	4024 (52.6)	142	3.1 (2.5, 3.7)
60–69	2226 (27.9)	169	6.4 (5.4, 7.5)
70–79	1251 (14.0)	222	15.9 (13.8, 18.2)
80 or more	493 (5.5)	152	29.8 (25.5, 34.4) *P-* trend < 0.001
Sex			
Female	4317 (51.9)	392	7.6 (6.8, 8.5)
Male	3677 (48.1)	293	6.8 (6.0, 7.7)
Education			
None	1324 (16.7)	185	11.8 (10.0, 13.8)
Elementary	4308 (55.5)	373	7.3 (6.5, 8.1)
High school	1723 (21.1)	92	4.7 (3.8, 5.8)
Higher education	569 (6.8)	16	2.1 (1.2, 3.7)
Subjective economic background			
Poor	2072 (31.0)	135	6.5 (5.5, 7.7)
Medium	3024 (42.3)	194	6.3 (5.2, 7.5)
Rich	1789 (26.7)	112	6.8 (6.0, 7.8)
Rural	3541 (48.0)	334	7.9 (7.0, 8.9)
Urban	4453 (52.0)	351	6.6 (5.8, 7.4)
Region			
Sumatra	1659 (20.8)	130	6.6 (5.5, 7.8)
Java	4647 (58.1)	309	6.4 (5.7, 7.2)
Main island groups^b^	1688 (21.1)	246	14.8 (13.1, 16.7)
Social capital (low)	1467 (20.8)	134	9.1 (7.8, 10.7)
Health variables			
Self-rated health (Unhealthy)	2849 (32.9)	335	9.9 (8.8, 11.2)
Body mass index			
Normal	2856 (39.0)	258	7.4 (6.4, 8.4)
Underweight	1063 (14.1)	145	11.7 (9.7, 13.9)
Overweight	1177 (15.8)	85	5.6 (4.4, 7.0)
Obese	2355 (31.1)	98	3.8 (3.0, 4.7)
Functional disability	2639 (31.3)	490	12.6 (11.3, 14.0)
Tobacco use			
Never	4663 (56.9)	371	6.6 (5,9, 7.4)
Quit	832 (9.8)	103	11.5 (9.3, 14.0)
Current	2499 (33.3)	211	7.1 (6.1, 8.2)
Diabetes	505 (6.3)	31	5.3 (3.6, 7.7)
Arthritis	1037 (11.8)	108	8.1 (6.6, 10.0)
Asthma	284 (3.4)	34	10.1 (7.0, 14.3)
Depression symptoms	1166 (17.0)	74	6.3 (5.1, 7.9)
Fruit (< 3 days/week) and vegetable consumption (<daily/week)	2119 (33.5)	141	6.7 (5.7, 7.8)
Hypertension	4391 (58.5)	417	9.5 (8.7, 10.4)
Heart attack, coronary heart disease, angina, or other heart problems	299 (3.6)	29	9.1 (6.1, 13.4)
Stroke	243 (2.9)	31	13.2 (9.0, 18.9)

^a^The analysis was adjusted considering the survey design of the study

^b^Major Island groups: Bali, West Nusa Tenggara, South Kalimantan, and South Sulawesi

### Associations with edentulism

In adjusted Poisson regression analysis, older age, living in five major island groups and having functional disability were associated with edentulism. In addition, among men, having quit and current tobacco use and among women, having low social capital were associated with edentulism. In further adjusted age stratified analysis, among 50 to 64 year olds, being male was negatively (Adjusted Prevalence Ratio: APR = 0.60, CI = 0.37, 0.98) and having underweight as positively (APR = 1.85, CI = 1.20, 2.83) associated with edentulism (see Table 2).

**Table 2:** Associations with edentulism estimated by Poisson regression

	All	Men	Women
Variable	Adjusted Prevalence Ratio^a^ (95% CI)	Adjusted Prevalence Ratio^a^ (95% CI)	Adjusted Prevalence Ratio^a^ (95% CI)
Sociodemographics			
Age in years			
50–59	1 (Reference)	1 (Reference)	1 (Reference)
60–69	2.21 (1.68, 2.91)***	2.38 (1.55, 3.68)***	2.13 (1.52, 3.00)***
70–79	4.89 (3.65, 6.56)***	4.92 (3.10, 7.79)***	5.05 (3.52, 7.25)***
80 or more	5.94 (3.76, 9.39)***	7.91 (4.27, 14.64)***	4.33 (2.08, 9.01)***
Sex			
Female	1 (Reference)	–	–
Male	0.83 (0.63, 1.10)		
Education			
None	1 (Reference)	1 (Reference)	1 (Reference)
Elementary	1.20 (0.88, 1.64)	1.00 (0.58, 1.72)	1.28 (0.86, 1.78)
High school/Higher education	0.88 (0.59, 1.30)	0.86 (0.46, 1.63)	0.79 (0.48, 1.32)
Subjective economic background			
Poor	1 (Reference)	1 (Reference)	1 (Reference)
Medium	1.12 (0.88, 1.43)	1.12 (0.79, 1.59)	1.15 (0.83, 1.59)
Rich	1.27 (0.95, 1.69)	1.40 (0.91, 2.16)	1.19 (0.82, 1.73)
Urban residence (base = rural)	1.10 (0.88, 1.38)	0.98 (0.70, 1.35)	1.28 (0.94, 1.73)
Region			
Sumatra	1 (Reference)	1 (Reference)	1 (Reference)
Java	0.77 (0.59, 1.00)	1.14 (0.74, 1.75)	0.56 (0.40, 0.79)***
Main island groups	2.08 (1.59, 2.71)***	2.75 (1.79, 4.21)***	1.74 (1.24, 2.45)***
Social capital (low)	1.16 (0.92, 1.47)	1.00 (0.68, 1.47)	1.32 (1.00, 1.79)*
Health variables			
Self-rated health (Unhealthy)	1.07 (0.86, 1.36)	1.17 (0.83, 1.65)	0.97 (0.73, 1.29)
Body mass index (Underweight)	1.23 (0.93, 1.62)	1.26 (0.85, 1.86)	1.18 (0.80, 1.75)
Functional disability	1.34 (1.08, 1.66)**	1.22 (0.89, 1.68)	1.40 (1.05, 1.87)*
Tobacco use			
Never	1 (Reference)	1 (Reference)	1 (Reference)
Quit	1.25 (0.86, 1.82)	1.95 (1.12, 3.39)*	0.73 (0.27, 1.92)
Current	1.17 (0.87, 1.56)	1.64 (1.01, 2.69)*	0.89 (0.51, 1.55)
Diabetes	0.56 (0.31, 1.01)	0.30 (0.09, 1.02)	0.78 (0.40, 1.52)
Arthritis	0.93 (0.69, 1.24)	0.80 (0.47, 1.38)	0.97 (0.69, 1.38)
Asthma	0.89 (0.52, 1.51)	0.45 (0.16, 1.29)	1.26 (0.69, 2.31)
Depression symptoms	0.73 (0.45, 1.16)	0.69 (0.34, 1.39)	0.74 (0.40, 1.39)

*CI* confidence interval; ***P < 0.001; **P < 0.01; *P < 0.05

^a^Pearson goodness-of-fit test > 0.9 for all models

### Associations between edentulism and health outcomes

In order to estimate the independent association between edentulism and 13 individual health outcomes, 13 separate multivariable models are calculated with edentulism as predictor and each health outcome as dependent variable. In adjusted analysis, among men, edentulism was positively associated with hypertension and negatively associated with diabetes and among women, edentulism was associated with functional disability (see Table 3).

**Table 3:** Associations between edentulism and health variables (outcomes) estimated by logistic regression

Health outcome	Men		Women	
	COR	AOR^a,b^	COR	AOR^a,b^
Health status (unhealthy)	2.30 (1.78, 2.97)***	1.18 (0.80, 1.75)	1.98 (1.58, 2.48)***	1.11 (0.80, 1.51)
Functional disability	2.40 (1.83, 3.84)***	1.36 (0.96, 1.92)	3.30 (2.59, 4.20)***	1.43 (1.05, 1.95)*
Underweight (vs. normal weight)	2.11 (1.51, 2.95)***	1.33 (0.83, 2.15)	1.92 (1.37, 2.69)***	1.16 (0.72, 1.88)
Overweigh or obesity (vs. normal weight)	0.48 (0.35, 0.67)***	0.64 (0.40, 1.01)	0.50 (0.38, 0.66)***	0.83 (0.58, 1.17)
Infrequent fruit and vegetable consumption	1.02 (0.74, 1.39)	1.02 (0.71, 1.47)	0.86 (0.64, 1.16)	0.96 (0.68, 1.34)
Current tobacco use	0.89 (0.69, 1.16)	1.15 (0.76, 1.75)	2.16 (1.44, 3.24)***	1.01 (0.55, 1.85)
Hypertension	2.40 (1.81, 3.17)***	1.74 (1.17, 2.57)**	2.00 (1.54, 2.58)***	0.93 (0.62, 1.39)
Heart problems^c^	2.04 (1.07, 3.88)*	1.09 (0.69, 5.17)	1.16 (0.65, 2.07)	1.05 (0.48, 2.31)
Stroke	2.72 (1.43, 5.15)**	0.89 (0.25, 3.18)	2.90 (1.02, 5.19)***	1.35 (0.31, 5.87)
Asthma	2.16 (1.25, 3.72)**	0.58 (0.21, 1.58)	1.40 (0.81, 2.44)	1.35 (0.68, 2.70)
Arthritis	1.29 (0.84, 1.97)	0.88 (0.50, 1.56)	1.58 (1.18, 2.10)**	0.98 (0.66, 1.44)
Diabetes	0.61 (0.29, 1.32)	0.29 (0.09, 0.98)*	1.26 (0.78, 2.03)	0.92 (0.47, 0.82)
Depression	0.56 (0.26, 1.18)	0.68 (0.31, 1.46)	0.70 (0.39, 1.25)	0.67 (0.34, 1.37)

*COR* crude odds ratio, *AOR* adjusted odds ratio

^a^Adjusted for age, education, socioeconomic status, rural-urban, region, social capital and all other health variables, as shown in this table

^b^Hosmer-Lemeshow goodness-of-fit test between predicted and observed probabilities was for all models *P* > 0.05

^c^Heart attack, coronary heart disease, angina, or other heart problems

***P<0.001; **P<0.01; *P<0.05

## Discussion

The study found in a national community-based survey among individuals 50 years and older, a 7.2% self-reported prevalence of edentulism in 2014/15 in Indonesia, which is similar to a measured survey of 17.6% in 2007 in Indonesia in individuals 65 years and older [7] (in this study 15.7% in the 65 years and older age group) and to what was found previously in China and South Africa [2]. The found prevalence rate of edentulism in Indonesia was higher than previously found in Ghana, Laos, Myanmar and in Vietnam, and lower than in India, Malaysia, Mexico, Philippines and Russia and the global prevalence of 14.0% [1, 2, 15]. The lower than global prevalence rate of edentulism in this study in Indonesia may be attributed to a lower consumption of non-refined carbohydrates and consequently lower dental caries and eventually tooth loss [1, 46]. The study found a significantly higher prevalence of edentulism in the five major island groups (Bali, West Nusa Tenggara, South Kalimantan, and South Sulawesi) than in Java and Sumatra. Reasons for this are not clear. It is possible that in the five major island groups greater gaps in dental care access exists than in Java and Sumatra.

The study found, in agreement with previous studies [2, 15, 47–50], that increasing age and in bivariate analysis having no education were associated with edentulism. It is possible that having no education translates into lower oral health knowledge and lower oral health services utilization where available [46]. Some previous studies [2, 48, 49] found a preponderance of edentulism among women and those living in rural areas, while in this study overall no significant gender and urban-rural differences were found. However, in age stratified analysis, among 50 to 64 year-olds, women had a higher prevalence of edentulism than men. Lower economic status was in this study not associated with edentulism, as found in some previous other studies [2, 48, 51]. This may be because economic status was assessed with a subjective measure, rather than an objective measure. Low social capital was in this study among women associated with edentulism. This finding is consistent with previous studies [20, 52]. It is possible that persons that belong to social networks are more likely to follow health-enhancing behaviours and, hence, have better health and oral health [53–55]. Previous research also showed that the effect of edentulism on facial appearance, eating and speech may decline in social capital due to embarrassment [1, 56].

Previous studies [2, 18, 48, 57] found an association between former and current tobacco use and edentulism, which was confirmed in this study among men. Edentulism was also associated with former smokers in China [15]; China and Indonesia have the highest prevalence of tobacco use among men in the world. Smoking is a known risk factor for periodontitis and tooth loss, meaning that the higher prevalence of edentulism among tobacco users may be directly related to the negative effects of tobacco use on periodontal health [18, 57]. Functional disability was found in this study to be both, a risk factor and a consequence of edentulism, which has also been found in previous studies [2, 12, 35, 36]. It is possible that people with a functional disability are less likely visit dental health care services [12]. Unlike in some previous studies [12, 17, 22], this study did not find an association between poor self-rated health as a risk factor for and consequence of edentulism. Arthritis, asthma, diabetes, angina and depression were in this study not associated with edentulism, as this was found in some previous studies [2, 11, 18, 19].

Among having different chronic conditions, this study found that edentulism was associated with hypertension. Similar results were found in previous studies [30, 31]. It is possible that total tooth loss is a risk factor for developing hypertension, which may be attributed to dietary changes of not be able to consume nutritious foods [30].

Some studies [11, 25–27, 58] found a significant association between undernutrition as a risk factor and consequence of edentulism, both of which were confirmed in this study in bivariate analysis, and in age stratified adjusted analysis, among 50 to 64 year-olds, having underweight was associated with edentulism. In a study among older adults in care homes in Indonesia it was found that individuals who were underweight had a significantly lower number of functional tooth units than those with normal weight [38]. The loss of teeth changes digestive processes and food choices contributing to nutritional deficiencies [38, 58]. Contrary to some previous studies [26–29, 59], this study found that edentulism was negatively associated with overweight or obesity in bivariate analysis. It is possible that edentulism caused individuals in this study to alter their diet, resorting to a diet that is high in fiber and low in saturated fat, reducing the risk of being obese. Contrary to some studies [23, 24], this study did not find that edentulism had a negative impact on fruit and vegetable consumption.

Contrary to some previous studies [24, 32–34], this study did not find an association between having edentulism and heart problems, stroke, asthma, arthritis, diabetes and depression. In a study in Indonesia the prevalence and severity of periodontitis (and consequently tooth loss) was not associated with arthritis [60] and among elderly in Indonesia, tooth loss was not associated with diabetes and heart diseases [10]. The diet and dietary habits of edentulous individuals in Indonesia may not be putting them at increased risk of heart diseases and diabetes [2]. It is also possible, since in this study arthritis, asthma, diabetes, and heart diseases were assessed by self-reported diagnosed conditions; they were underreported and thus contributing to these findings [2]. Sudiono [10] proposes that among elderly in Indonesia the loss of teeth may have been influenced by “a low demand for dental hygiene” and that “improvement of dental awareness and education in oral hygiene measures in such populations might reduce both diseases (dental caries and periodontitis) responsible for tooth loss and could improve quality of life in Indonesia.”

### Study limitations

Edentulism was only measured with one question. Oral examinations should be conducted in future investigations. The data analysed were based on a cross-sectional survey, so no causative conclusions be drawn between independent study variables and the prevalence of edentulism. Finally, the analysis was limited to the variables included in IFLS-5, and other factors such as inadequate oral hygiene and dental attendance found significant in previous investigations should be included in future research.

## Conclusions

Almost one in ten older adults (50 years and above) Indonesians were edentate. Results suggest that overall and/or among men or women that older age, living in five major island groups, having functional disability, tobacco quitters and users and those with low social capital was associated with edentulism. The identified associated factors of edentulism may be utilized in oral health programmes targeting older adults in Indonesia.

## Abbreviations

BMI: Body Mass Index
CES-D: Centres for Epidemiologic Studies Depression Scale
DBP: Diastolic Blood Pressure
EA: Enumeration Area
IFLS: Indonesian Family Life Survey
SBP: Systolic Blood pressure
